# Influence of fibrinogen and C-RP on progression of peripheral arterial disease in type 2 diabetes: a preliminary report

**DOI:** 10.1186/1475-2840-12-29

**Published:** 2013-02-01

**Authors:** Marijan Bosevski, Golubinka Bosevska, Lily Stojanovska

**Affiliations:** 1 Medical Faculty, University Cardiology Clinic, Skopje, Macedonia; 2 Institute for Public Health of R. Macedonia, Skopje, Macedonia; 3 College of Health and Biomedicine, Victoria University, Melbourne, Australia; 4 School of Biomedical and Health Sciences, Victoria University, PO Box 14428, 8001, Melbourne, Vic, Australia

**Keywords:** ABI, Fibrinogen, C-RP, Type 2 diabetes, Peripheral arterial disease, Progression of atherosclerosis

## Abstract

**Background:**

Limited studies have suggested that inflammatory biomarkers play a role in the initiation and progression of atherosclerosis in diabetic patients. This study assesses the effect of inflammatory biomarkers: fibrinogen and C-reactive protein (C-RP) on the progression of peripheral arterial disease (PAD) in type 2 diabetic (T2D) patients.

**Methods:**

Sixty two patients with T2D and PAD (mean age 60.28 ± 27 years and diabetes duration of 8.58 ± 6.17 years) were enrolled in a cohort prospective study of 36 months. Ankle-brachial index (ABI) was measured in all patients at baseline and after 36 months. Multiple linear regression analysis was used to determine the predictivity of variables for fibrinogen, C-RP, plasma lipid fractions, fasting plasma glucose, Body Mass Index (BMI), duration of diabetes status and the age on changes in ABI value.

**Results:**

Linear regression analysis defined F as a predictor for endpoint value of ABI (β = 0.469, p = 0.007). Value of C-RP determinates change of minimal value of ABI (β = 0.449, p = 0.037) and change of mean ABI per year (β = 0.442, p = 0.025).

**Conclusion:**

Our data indicate that plasma determination of fibrinogen and C-RP might have a clinical implication in defining the process of progression of PAD in T2D population.

## Background

Previous studies have found accelerated atherosclerosis and increased risk of vascular disease in diabetic patients [1,2]. Risk factors such as hyperglycaemia and glucose intolerance, hyperlipidaemia, obesity and hypertension have been established as risk factors for diabetic vascular disease [3,4].

However, very little is known about the potentially unique features of this inflammatory process in diabetic vascular disease. Some studies have suggested that the inflammatory biomarkers, C-RP and fibrinogen, play a role in initiation, aggravation of the classical pathways and progression of atherosclerosis in diabetic patients [5-7]. These biomarkers are more closely related with the metabolic syndrome and insulin resistance compared with cytokines, and they influence the onset of potential cardiovascular events [8].

Hyperfibrinogenemia, a condition of elevated level of fibrinogen in the blood, is found more frequently in diabetic patients with manifested peripheral arterial disease (PAD) and more severe coronary artery disease (CAD) [9]. High levels of C-RP have been found in advanced stages of atherosclerosis in diabetic patients, especially in those with high level of HbA1C and high concentration of advanced glycated proteins [10]. The purpose of this study was to determinate the influence of inflammatory biomarkers: fibrinogen and C-RP on progression of PAD in type 2 diabetic (T2D) patients as measured with changes in ABI values.

## Methods

Sixty seven patients with T2D and PAD were enrolled in a cohort prospective study between 2005 and 2008. Five patients were excluded from the study, while the remaining 62 patients that met the inclusion criteria were followed-up for 36 months. The study was conducted at the vascular laboratory at University Cardiology Clinic Skopje. The study was carried out according to the Helsinki declaration and was approved by the University Clinic Ethics Committee, Skopje.

Type 2 diabetes was defined based on the criteria of the International Diabetes Federation. Patients with PAD, stage Fontaine I, with an established value of ABI < 0. 9 met the inclusion criteria for the study. Those with advanced PAD stage and with high, pathological ABI values, as well with acute inflammatory state (diabetic foot infection, cold, pneumonia) were excluded from the study. In all patients, we measured at baseline and after 36 months, at the completion of the study the ABI value, using a continuous wave Doppler (Hunleigh Healtcare, Cardiff, UK) to determine the lowest ABI values (ratio of ankle to brachial pressure). ABI was measured by vascular physicians with the same person being involved throughout the study. Inter-observer variability was up to 6%, measured previously. Mean values were calculated from the average of two measurements. Following the endpoint measurement of the ABI, a change in ABI was also calculated.

Laboratory tests were conducted at the University Institute for Clinical Biochemistry. Fibrinogen and C-RP were determined using the BNII nephelometer (N High-Sensitivity C-RP and N Antiserum to Human Fibrinogen; Dade Behring). Serum levels of the following laboratory tests were measured at baseline and at 36 weeks in all patients: total cholesterol (enzymatic methods-in the presence of cholesterol oxidize), triglycerides (in the presence of glycerokinase), and the HDL fraction by direct method.

Automatic analyzer Coba Integra 400/700 (ROCHE Diagnostics) was used. The LDL fraction was evaluated using the Friedewald formula. Non-HDL cholesterol was determinate as a value of total cholesterol minus HDL cholesterol. Fasting plasma glucose concentration was evaluated using the enzymatic-photometric method, in the presence of glucoso-dehydrogenase. All analyses were done according recommendation of International Federation of Clinical Chemistry and Laboratory Medicine.

Multiple linear regression analysis was used to define continuous variables with predictive value for the ABI, when adjusted for systolic blood pressure, BMI index, diabetes duration, age, blood glucose, plasma lipid levels (total, LDL-, HDL- cholesterol), C-RP and fibrinogen. The data is expressed as mean ± SD.

## Results

Sixty-two participants were included for analysis, comprising 39 males and 23 females. The baseline characteristics for the study population were as follows: mean age of 60.28 ± 27 years; mean diabetes duration of 8.58 ± 6.17 years, and overweight status by BMI (mean BMI 28.7 + 4 kg/m^2)^.

At baseline the mean fasting plasma glucose of the group was 8.5 ± 2.4 mmol/L, mean plasma fibrinogen level was 4.12 ± 0.85 g/L and had a mean of C-RP of 5.69 ± 1.92 mmol/L. Measurements of the lipid profile at baseline show total cholesterol of 5.4 ± 1.4 mmol/L, LDL-cholesterol of 3.3 ± 0.9 mmol/L, and HDL-cholesterol of 1.0 ± 0.4 mmol/L.

Of all the patients 98.4% received acetyl salicylic acid, while 85.4% received statins. Furthermore, 59.6% of the patients were treated with oral anti-diabetics and 41.4% of them with insulin.

The measurements of the ABI indexes at baseline were: ABIx = 0.83 ± 0.04 (mean) and ABImin = 0.75 + 0.07 (lowest), while following the 36 months we found ABIx = 0.45 ± 0.05 and ABImin = 0.48 ± 0.06. Significant changes of minimal value of ABI (dABImin = 0.38 ± 3.1) and mean ABI (dABIx = 0.27 ± 0.21) were found. No gender differences were found according ABI indexes.

Multivariate analysis showed that F value has been determined with non HDL - cholesterol (β = 1.093, p = 0.027). Linear regression analysis defined fibrinogen as a predictor for minimal value of ABI, found at the completion of the study (β = 0.469, *p* = 0.007).

**Figure 1:**
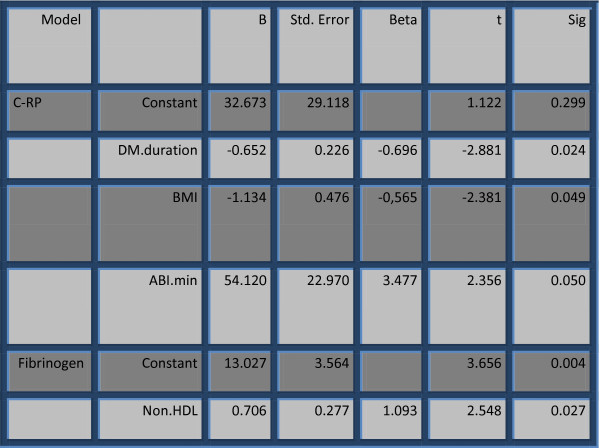
Multiple linear regression analysis for factors for C-RP and fibrinogen.

The value of C-RP was independently determined with diabetes duration, BMI and minimal value of ABI, according to the results of multiple linear regression analysis. Fibrinogen was determinated with the value of non HDL - cholesterol (Figure 1).

**Figure 2:**
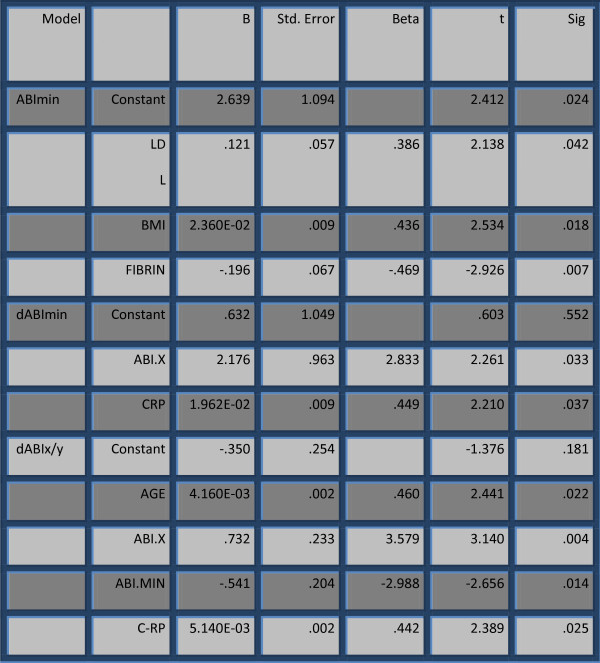
C-RP and Fibrinogen as factors for progression of ABI (multiple linear regression analysis).

Linear regression analysis defined fibrinogen as predictor for minimal value of ABI, found at the end completion of the study (β = 0.469, p = 0.007). The value of C-RP determinates the change of minimal value of ABI (dABImin) and the change of mean ABI per year (dABIx/y) (Figure 2).

## Discussion

Patients with T2D often present with hypercoagulability, which may be related to changes in inflammatory biomarkers [11]. Patients with vascular disease and T2D presented higher values of inflammation markers when compared with patients without vascular disease [12]. In our study, both biomarkers, C-RP and fibrinogen, were significantly elevated.

### Impact of fibrinogen

Fibrinogen and non-HDL cholesterol have synergistic effects, as factors accelerating the progression of carotid atherosclerosis [13]. Fibrinogen increases atherogenic dislipidaemia in diabetic patients. Fibrates act synergistically in the regulation of dispilidaemia, reduction in fibrinogen, triglycerides, LDL and non HDL- cholesterol [14,15]. Increased fibrinogen, as an inflammatory, haemostatic component, is present in diabetic patients, who are on oral hypoglycaemic as well as on insulin therapy [16].

Studies have shown that fibrinogen is associated with diabetes regulation, age, hypertension and components of the metabolic syndrome [17,18].

Increased fibrinogen levels are often present in diabetic patients with PAD, and there is a correlation with fibrinogen values and angiographic extent of the disease [19,20]. Fibrinogen compared with C-RP might be closely associated with diabetic vascular disease [21]. The minimal value of ABI, found at the completion of our study correlates with the levels of fibrinogen measured at baseline. Fibrinogen values over 3.5 g/L have been shown previously to be an independent marker for progressive cardiac events in diabetic patients [22]. Our results show that increased fibrinogen level of >4 g/L is a risk factor for progression of PAD, as assessed by a decrease of ABI value.

Some studies have implied that the process of inflamation is the bridge between metabolic syndrome, insulin resistance and atherosclerosis [23]. As the latter causes are highly prevalent in diabetic patients, it is expected to observe a presence of significant inflamation in diabetic patients. Inflamation influences development of prediabetic phase in type 2 diabetes mellitus [24].

### C-RP in diabetic vascular disease

According to our results the C-RP value is independently connected with diabetes duration, BMI and ABI index. Obesity is a major risk factor, correlated with C-RP in populations with metabolic syndrome and/or T2D (*ADOPT*) [25,26]. Follow up studies in diabetic patients with coronary and carotid atherosclerosis, have shown that advanced atherosclerosis in T2D is connected with increased C-RP values and increased concentrations of advanced glycosylation products [27]. This high cardiovascular risk may be due to a combination of non-classical risk factors associated with insulin resistance, e.g. inflammation, hyperinsulinemia, oxidative stress, and hypercoagulability, together with the separate components of metabolic syndrome [28,29].

Serum C-RP level was independently associated with PAD in T2D in a cross-sectional study by Yu and associates [30]. Our longitudinal study has shown that C-RP is an independent factor for dynamic change of minimal and mean values of ABI.

### Therapeutic possibilities

Potential applications of these observations have implications in clinical management of T2D patients with PAD. Previous studies imply that measuring inflammatory biomarkers may improve the definition of cerebrovascular risk profile in patients with type 2 diabetes [31]. Improvement of inflammatory state in diabetic population is associated with improvement of glyco-metabolic control. The use of insulin, metformin, sulfonylurea, thiazolindiones, and especially high doses of statins, according to the above studies, lead to reduction in C-RP and other inflammatory biomarker values in patients with T2D [32-36]. The use of statins, fibrates and glitazones although favorable, are not sufficient to cause the progression of diabetic vascular disease [37,38].

### Limitations

ABI is defined as reliable marker for definition PAD, despite numerous factors that influence its value [39]. We measured progression of peripheral atherosclerosis using this index.

Our results are of a preliminary nature and as such this study has limitations due to its small sample size. Larger prospective studies in the future are therefore necessary.

## Conclusion

Our results indicate that fibrinogen and C-RP independently influence the progression of peripheral atherosclerosis in T2D patients. Plasma determination of these inflammatory biomarkers might have an implication in predicting the clinical course of peripheral arterial disease in T2D population.

## Abbreviations

T2D: Type 2 Diabetes;PAD: Peripheral arterial disease;ABI: Ankle-brachial index;C-RP: C-Reactive protein

## Competing interests

The authors declare that they have no competing interests.

## Authors’ contribution

MB: design and results of study. GB: contributed to biomarker analysis and related discussion. LS: revised the manuscript critically for important intellectual content. All authors read and approved the final manuscript.
